# Diagnostic value of two-time point [^68^Ga]Ga-PSMA-11 PET/CT in the primary staging of untreated prostate cancer

**DOI:** 10.1038/s41598-023-35628-0

**Published:** 2023-05-22

**Authors:** Friedrich Weitzer, Birgit Pernthaler, Elisabeth Plhak, Regina Riedl, Reingard Maria Aigner

**Affiliations:** 1 Division of Nuclear Medicine, Department of Radiology, Medical University of Graz, Auenbruggerplatz 9A, 8036 Graz, Austria; 2 Institute for Medical Informatics, Statics and Documentation, Medical University of Graz, Auenbruggerplatz 2, 8036 Graz, Austria

**Keywords:** Metastasis, Cancer, Prostate cancer, Cancer, Prostatic diseases

## Abstract

The emerging PET tracer [^68^Ga]Ga-PSMA-11 has been established for staging in prostate cancer (PCa). Aim was to determine the value of early static imaging in two-phase PET/CT. 100 men with newly diagnosed histopathologically confirmed untreated PCa who underwent [^68^Ga]Ga-PSMA-11 PET/CT from January 2017 to October 2019 were included. The two-phase imaging protocol consisted of an early static scan of the pelvis (6 min p.i.) and a late total-body scan (60 min p.i). Associations of semi-quantitative parameters derived via volumes of interest (VOI) with Gleason grade group and PSA were investigated. In 94/100 patients (94%) the primary tumor was detected in both phases. In 29/100 patients (29%) metastases were detected at a median PSA level of 32.2 ng/ml (0.41–503 ng/ml). In 71/100 patients (71%) without metastasis a median PSA level of 10.1 ng/ml (0.57–103 ng/ml) was observed (*p* = < 0.001). Primary tumors demonstrated a median standard uptake value maximum (SUVmax) of 8.2 (3.1–45.3) in early phase versus 12.2 (3.1–73.4) in late phase and a median standard uptake value mean (SUVmean) of 4.2 (1.6–24.1) in early phase versus 5.8 (1.6–39.9) in late phase, significantly increasing over time (*p* = < 0.001). Higher SUVmax and SUVmean were associated with higher Gleason grade group (*p* = 0.004 and *p* = 0.003, respectively) and higher PSA levels (*p* = < 0.001). In 13/100 patients the semi-quantitative parameters including SUVmax were declining in the late phase compared to early phase. Two-phase [^68^Ga]Ga-PSMA-11 PET/CT demonstrates a high detection rate for primary tumor of untreated PCa of 94% and improves diagnostic accuracy. Higher PSA levels and Gleason grade group are associated with higher semi-quantitative parameters in the primary tumor. Early imaging provides additional information in a small sub-group with declining semi-quantitative parameters in the late phase.

## Introduction

Prostate cancer (PCa) is the most common cancer found in older men worldwide, contributing significantly to mortality and morbidity. Despite good outcome in localized disease, advanced metastatic disease generally remains incurable. Lethality in advanced disease is of major concern since there is a lack of long-term therapeutic response despite significant improvements in understanding tumor genetics, tumor environment, intrinsic and extrinsic tumor mechanisms, and technical advances in disease detection^1^.

Current urological guidelines concerning diagnosis and treatment of PCa emphasize classifying the patients into low, medium, and high risk groups as an essential part of staging for treatment options and outcome. Risk stratification is based on digital rectal examination, prostate specific membrane antigen level (PSA), the histopathological report including Gleason grade group (GG) and amount of tumor mass, as well as imaging methods. Conventional imaging methods such as contrast-enhanced CT and MRI are considered insufficient due to relying on non-specific size criteria^2^.

Prostate specific membrane antigen (PSMA) positron emission tomography/computed tomography (PET/CT) is an emerging diagnostic tool in nuclear medicine reported with promising results in the setting of metastatic PCa as well as in biochemical recurrence (BCR) of PCa^3^. PSMA is highly expressed in up to 98–99% of all forms of PCa with significantly increasing expression linked to metastatic disease, recurrence and biological tumor aggressiveness^2^.

Over-expression of PSMA is found in over 90% of prostate cancer, offering a viable target for molecular imaging, including PET ligands. [^68^Ga]Ga-PSMA-11 PET/CT has proven as a reliable diagnostic tool in staging prostate cancer and has become the standard diagnostic method for staging newly diagnosed prostate cancer in some countries, replacing previously used CT^4^. Various PSMA ligands are currently available, labelling includes Technetium-99 m, Gallium-68, Copper-64 and Fluorine-18 for diagnostic purposes^5–7^. Additionally, Lutetium-177, Copper-67 and Actinium-225 labelled PSMA ligands are currently investigated for therapeutic approach^8,9^. PSMA ligands offer the ability for a true theranostic approach when labelled with beta-emitters as Lutetium-177 or Copper-67^8,9^ while experimentally used alpha-emitters as Actinium-225 show remarkable effects in advanced-stage patients ^10,11^.Recent urological guidelines as the updated 2020 EAU-EANM-ESTRO-ESUR-SIOG guideline highlight the improved sensitivity for detecting nodal and distant metastasis although the clinical value of the improved sensitivity is not known yet. A recent update in March 2023 emphasizes the usage of PSMA PET in primary staging over CT and MRI especially for nodal metastasis, however the clinical value for patient outcome is still unknown. This is a significant change to the 2017 version which did not mention PSMA PET/CT scan for primary staging in PCA as it was still seen in the experimental stage^12–15^. Although study design in PSMA PET/CT is often centered on screening of metastasis in biochemical recurrence of prostate cancer, a retrospective study reported a high negative predictive value in high-risk PCa for local lymph node metastasis^16^. Furthermore, early detection of metastasis can lead to a radical change in therapy in up to 50% of cases, as described in a prospective multicenter study^17^.

Study aim was to evaluate the diagnostic and clinical value of semi-quantitative parameters in the PCa in the primary tumor including maximum standard uptake value (SUVmax), mean standard uptake value (SUVmean) and total tumor volume (TTV) by using a two-time point [^68^Ga]Ga-PSMA-11 PET/CT protocol consisting of early and late static scans.

## Methods

This retrospective study included 100 patients from January 2017 to October 2019 with untreated PCa referred to our division for primary staging with [^68^Ga]Ga-PSMA-11 PET/CT. Patients were referred for primary staging after establishing diagnosis via trans-rectal biopsy and performing pre-staging via MRI and/or CT of the pelvis.

All procedures performed in this study were in accordance with the ethical standards of the institutional research committee (Ethikkommission der Medizinischen Universität Graz—Ethics Committee at Medical University of Graz). Being a retrospective study the need for written informed consent was waived by the Ethics Committee at Medical University of Graz.

The Gallium-68 labelled PSMA-11 ligand was prepared in-house according to the established EANM guideline on current good radiopharmacy practice (cGRPP)^18^ by the chelation of the HBED-CC precursor molecule (ABX, Radeberg, Germany) via a GMP-compliant fully automated radiopharmaceutical synthesizer (GRP Module, Scintomics, Fuerstenfeld-Bruck, Germany). Gallium-68 was obtained from a Germanium-68/Gallium-68 Generator (Galli Ad, IRE, Fleurus, Belgium). Quality control was successfully carried out according to the European Pharmacopeia. All prepared products were apyrogenic and sterile with a radiochemical purity > 91%.

All PET/CT examinations were performed on two dedicated PET/CT systems in 3D mode (Discovery MI, GE Healthcare, Milwaukee, WI, U.S.A.; Biograph mCT, Siemens, Erlangen, Germany); patients were unsystematically referred to each scanner. Target dose was 2 MBq kg body weight range 80–200 MBq according to established EANM dose recommendations^19^. The early phase consisted of a static image acquired 6 min post-injection over the pelvis with one bed position and an acquisition time of 2 min. The late phase consisted of static whole-body imaging starting 60 min post-injection by discontinuously craniocaudal bed movement and an acquisition time of 2 min per bed position. Transmission CT scans for attenuation correction were acquired using helical mode without the use of a contrast agent. Both PET and CT scans were reconstructed with a slice thickness of 3.75 mm. All studies were interpreted by at least two experienced Nuclear Medicine physicians in consensus reading, seldom discrepancies were solved by adding a third experienced Nuclear Medicine physician.

[^68^Ga]Ga-PSMA-11 positive tumor lesions were visually identified as focal uptake higher than adjacent background activity, not associated with physiological uptake as recommended by the generally accepted international EANM/SNMMI criteria^20^. Volumes of interest (VOI) to acquire SUVmax, SUVmean and TTV were manually drawn over pathological uptake in the prostate gland in both early and late images, avoiding the very high physiological bladder activity. VOIs were also drawn to acquire SUVmax, SUVmean and TTV in up to five extra-prostatic non-physiological uptakes in the whole-body late phase PET image to document nodal and bone metastasis.

Semi-quantitative data for both primary tumor and metastasis was acquired on AW Server 3.2 Ext. 4.0, GE Healthcare, Milwaukee, WI, U.S.A. using the manufacturer provided auto-snake tool with automatic threshold definition pre-set to 42% of SUVmax.

Risk stratification was based on the generally accepted criteria proposed by D’Amico in 1998^21^: The definition of (a) low risk are Gleason grade group 6, prostate specific antigen (PSA) level < 10 ng/mL, and clinical stage cT1c or cT2a, (b) intermediate risk are Gleason grade group 7 or PSA level 10-20 ng/mL or clinical stage cT2b, and (c) high risk are Gleason grade grou*p* ≥ 8 or PSA level > 20 ng/mL or clinical stage cT2c or higher. Patients with a history of previous or present other neoplastic disease were dismissed. Patients were included regardless of newly started anti-hormone treatment or pre-existing benign prostatic disorders. Anti-hormone treatment was started in all cases in less than a month before PET/CT examination, therefore a significant impact on results was deemed insignificant.

Therapy changes due to PET/CT outcomes were performed by the referring urologist; however complex cases and equivocal findings were discussed in the weekly tumor-board held for urological tumors held at the University Department of Urology including one nuclear medicine physician.

Statistical analysis was performed using SAS™ version 9.4 (SAS Institute Inc., Cary, NC, U.S.A.) by a professional statistician. Continuous parameters are presented as median, minimum and maximum, categorical parameters as frequency and percent. Wilcoxon signed-rank test was used to compare late and early phase semi-quantitative parameters. For group comparisons (i.e., PSA levels between patients with and without metastasis, semi-quantitative parameters between Gleason and D’Amico grading groups), Mann–Whitney-U-test and Kruskal–Wallis test were used. Additionally, Spearman’s rank correlation coefficient was calculated for PSA levels and semi-quantitative parameters.

### Ethics approval

This study was performed in line with the principles of the Declaration of Helsinki. Approval was granted by the Ethics Committee at the Medical University of Graz. Being a retrospective study the need for written informed consent was waived by the Ethics Committee at Medical University of Graz.

## Results

From January 2017 to October 2019 647 [^68^Ga]Ga-PSMA-11 PET/CT scans were performed at our division; out of these 128 patients were referred for primary staging. 28 patients were excluded due to insufficient clinical data, including nine patients where radical prostatectomy or follow-up biopsy revealed no malignancy. The final patient collective included 100 patients with histopathologically proven prostate cancer. Table 1 shows patient characteristics including final clinical TNM staging.

**Table 1:** Patient characteristics including Gleason grade group, PSA levels and D’Amico classification.

Patients	Total	n = 100
Median patient age (years)	69.51	(Range 50–83)
Initial Gleason grade group		
6	14	(14%)
7	34	(34%)
8	18	(18%)
9	26	(26%)
10	8	(8%)
Primary tumor stage (TNM)		
T1c	52	(52%)
T2a–c	22	(22%)
T3a–b	23	(23%)
T4	3	(3%)
N0	79	(79%)
N1	21	(21%)
M0	84	(84%)
M1	16	(16%)
PSA (ng/ml)		
Median PSA at diagnosis (ng/ml)	12.02	(Range 0.41–503)
< 10 ng/ml	40	(40%)
≥ 10 ng/ml	60	(60%)
Median PSA present nodal metastasis (ng/ml)	37.46	(Range 0.97–503)
Median PSA present bone metastasis(ng/ml)	35.24	(Range 0.41–503)
Median PSA absent metastasis (ng/ml)	10.1	(Range 0.57–103)
D’Amico classification		
Low	9	(9%)
Intermediate	29	(29%)
High	62	(62%)

**Figure 1:**
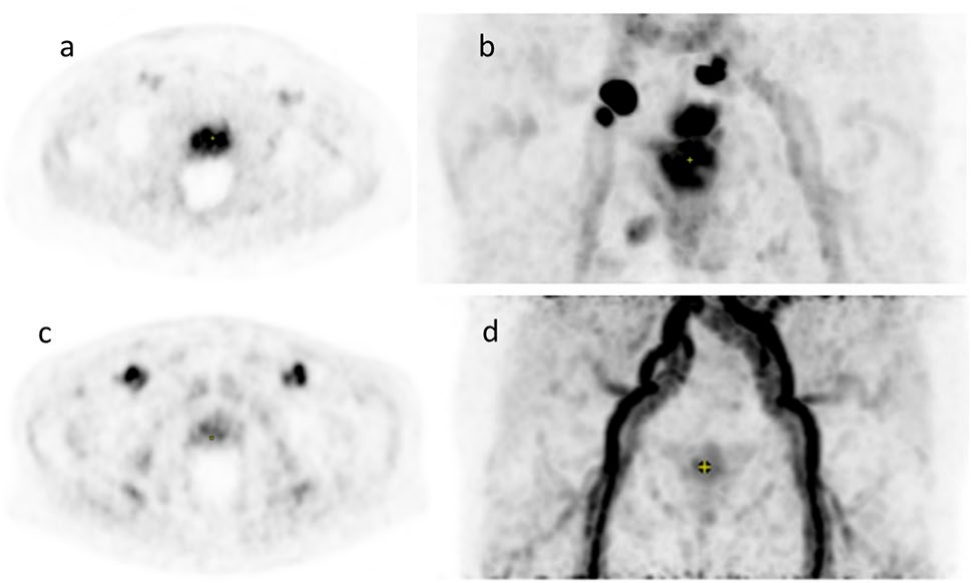
Visual comparison of two patients shows association between SUVmax levels and tumor stage. In the upper row (**a**, **b**) SUVmax was 27.2 and MTV 38.1 while in the lower row (**c**, **d**) SUVmax was only 9.6 and MTV 26.0. Patient on the upper row also presented with several pelvic lymph node metastasis, visible in MIP mode (**b**).

In 94/100 patients (94%), focal pathological uptake in the prostate gland representing the primary tumor could be detected in early and late phases. In the remaining 6/100 patients (6%) only diffuse non-suspicious [^68^Ga]Ga-SMA-11 uptake in the whole prostate gland indistinguishable from background was visible (see Fig. 1 for representative early phase).

[^68^Ga]Ga-PSMA-11 PET/CT revealed lesions highly suspicious for metastasis in 29/100 patients (29%). Nodal lymph node metastasis only was found in 13/100 patients (13%), bone metastasis only in 7/100 patients (7%), and combined nodal and bone metastasis in 9/100 patients (9%).

In 39/100 patients (39%), [^68^Ga]Ga-PSMA-11 PET/CT led to a substantial change in therapy. In 18/100 patients (18%) additional chemotherapy or combined radiation therapy or additional radiation therapy only was chosen. In 21/100 patients (21%) [^68^Ga]Ga-PSMA-11 PET/CT led to a down-staging and less aggressive therapy regime. 33/100 (33%) patients underwent radical prostatectomy, 67/100 (67%) anti-hormone treatment, 53/100 (53%) radiation therapy, and 15/100 (15%) conventional intensified chemotherapy.

PCa associated lesions demonstrated in the early versus late images a median SUVmax value of 8.2 (range 3.1–45.3) versus 12.2 (range 3.1–73.4) and a median SUVmean of 4.2 (range 1.6–4.1) versus 5.8 (range 1.6–39.9) both statistically significantly increasing over the time (*p* = < 0.001). (See Table 2) Consecutive analysis of early and late (partially) visible bone or nodal metastasis were not performed since a considerable bias to missing data from non-pelvic metastasis had to be taken in account.

**Table 2:** Chart displaying characteristics of SUVmax (= maximum standard uptake value) and SUV mean (mean standard uptake value) in early versus late images.

SUV	Mean	SD	Median	Minimum	Maximum	*p* value
SUVmax early	10.41	7.50	8.15	3.14	45.27	< 0.001
SUVmax late	18.88	15.41	12.20	3.10	73.41	
SUVmean early	5.31	3.32	4.16	1.61	24.11	< 0.001
SUVmean late	9.00	7.61	5.81	1.57	39.86	

SD, Standard deviation; *p* value, Wilcoxon test.

Further analysis revealed in 13/100 cases (13%) declining SUVmax values in the primary tumor from 10.73 to 7.19 (early range: 5.58–40.55, late rage: 3.1–22.32) while the standard deviation was declining from 8.98 in early images to 4.81 in late images. Primary tumor stages were varying from pT1c to pT3b, nodal metastasis were present in three cases while no distant metastasis was found in all 13 patients. Similar findings were found when comparing mean SUVmean (early 6.04 vs. late 4.12) TTV (early 31.07 vs. late27.1) and total lesion PSMA uptake (TLU) values (early 156.66 vs. late 78.65). Therefore, in a small sub-group with no obvious other conditions early images may provide additional information.

Patients who presented with metastasis in [^68^Ga]Ga-PSMA-11 PET/CT had significantly higher PSA levels (32.2 ng/ml, range 0.4–503.0 ng/ml) than patients without metastasis (10.1 ng/ml, range 0.6–102.9 ng/ml) (*p* =  < 0.001).

**Figure 2:**
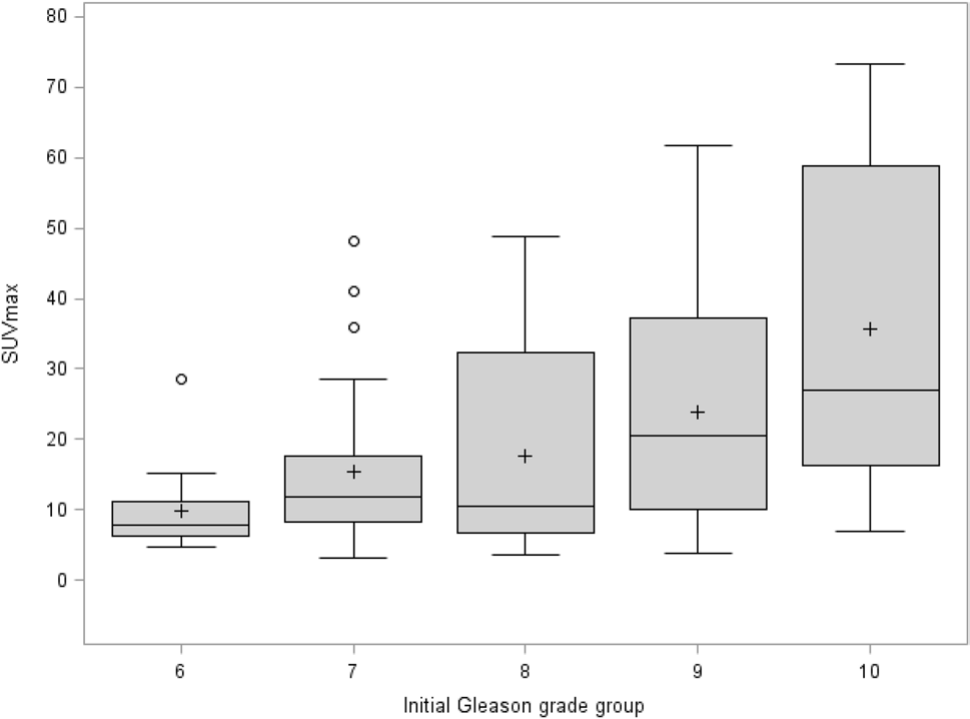
Box-plot display of SUVmax values correlating positively to Gleason grading.

Higher SUVmax and SUVmean were observed for higher Gleason grading (*p* = 0.004 and *p* = 0.003, respectively) and PSA levels positively correlated with SUVmax (Spearman’s rank correlation coefficient: r = 0.40, *p* =  < 0.001), SUVmean (r = 0.35, *p* < 0.001), and TLU (r = 0.34, *p* = 0.0004), but no for TTV (r = 0.12, *p* = 0.215) (see Fig. 2).

Risk stratification according to D’Amico criteria revealed a prominent proportion of high risk cases: high risk (n = 62), intermediate risk (n = 29), low risk (n = 9). Higher SUVmax and SUVmean levels were observed in higher D’Amico grading (*p* = 0.004 and *p* < 0.001, respectively), however there was no statistically significant association concerning TTV (*p* = 0.55) and TLU (*p* = 0.05).

## Discussion

Primary staging, correct early risk stratification, and detection of metastasis is essential for optimal first-line treatment in prostate cancer. PSMA PET/CT has proven to be an important step towards personalized medicine^1^ while showing a great influence in further patient management^16^ and demonstrating a greater sensitivity than conventional imaging^22^.

Although semi-quantitative parameters including both SUVmax and SUVmean levels are significantly increasing over time in early versus late images, early images are still useful for detecting small lesions before physiological tracer excretion in the bladder is present, possibly masking them. Early imaging may provide additional information since in a small sub-group (13/100 patients) the semi-quantitative parameters including SUVmax were declining in the late phase. No other common conditions or pre-test markers were identified in this sub-group in our study.

The time-point of 6 min p.i. for early imaging has already been described in a small study by Wen et al.^23^ containing 11 patients as optimal for SUVmean values in the prostate since urinary bladder activity rapidly increases after 5.5 min. Continuous upward trend for SUVmean values through 60 min was also present.

Our findings are consistent with findings reported in two similar smaller studies by Sachpekidis et al.^24,25^ using early dynamic imaging in a 24 frame protocol (23/24 positive patients and 12/16 positive patients vs. 94/100, detection rate 95.8% and 75% vs. 94%), although the small patient number in both studies presents a major limitation. SUVmax values were comparable to our study (10.2 vs. 12.2 in our study).

Hofmann et al.^26^ reported in a prospective, randomized, multicenter study a higher sensitivity for [^68^Ga]Ga-PSMA-11 PET/CT of 85% (94% in our study) compared to conventional imaging (27%). However, pelvic nodal (20%), abdominal nodal (9%) and bone metastasis were more prevalent than in our study (29% overall metastasis, 20% nodal, 9% combined nodal and bone metastasis).

Uprimny et al.^27^ reported similar findings using comparable early dynamic imaging for 8 min 60 s per frame (82/90 positive patients, 91.1% vs. 94/100 patients, 94% respectively. Median SUVmax values in the primary tumor were comparable to our study (12.5 vs. 12.2) while higher median SUVmax and SUVmean levels in two-phase [^68^Ga]Ga-PSMA-11 PET/CT in primary tumor lesions were associated with higher Gleason score in both studies. Detection rate of nodal and bone metastasis was also comparable (26.7% vs. 29%). Moreover, a positively correlation between PSA levels and SUVmax could be shown in both studies; therefore, higher SUVmax indicates a sub-group with higher PSA levels and higher Gleason grade group which both fall into the high-risk group.

Interestingly, a 2020 study by Hoffmann et al.^28^ reported a lower lesion positivity rate in both primary staging (13/18 patients, 72%) and restaging (147/215 patients, 68%), however the additional value of late imaging 3 h p.i. in a few individual cases. due to higher tracer-uptake and improved contrast was mentioned. Further base to this claim was delivered by Baukneht et al.^29^ using forced diuresis followed by late imaging. The authors of this prospective study concluded that systematic use of both methods could not be supported, although there may be a benefit of different time points in certain scenarios.

The correlation of higher SUVmax values with higher Gleason grade group and PSA levels are well known and backed up by similar findings by other authors^30^.

The 2020 updated EAU-EANM-ESTRO-ESUR-SIOG guideline and especially the recent march 2023 update^13–15^ see PSMA PET/CT is no longer in an experimental role compared to the older 2017 version although more studies concerning patient outcome are needed^12^. In up to 39/100 cases (39%) PET/CT findings let to a significant change in patient management, especially in 21/100 patients (21%) where down-staging and a less aggressive therapeutic approach was eligible. Since these measurements included non-invasive methods like radiotherapy and/or chemotherapy it was impossible to evaluate the sensitivity and specificity compared to the gold standard histopathology in diagnosing nodal and bone metastasis. Furthermore, patients underwent curative radical prostatectomy and pelvic lymphadenectomy in only 67/100 cases (67%) therefore the use of [^68^Ga]Ga-PSMA-11 PET/CT in combination with PSA levels could be proposed as gold standard for primary staging in prostate cancer. These findings show that despite the unclear role of PSMA PET/CT and it’s increased sensitivity in current urological guidelines, [^68^Ga]Ga-PSMA-11 PET/CT is already used as diagnostic tool for primary staging and therapy decision of prostate cancer by clinicians.

A limitation of this retrospective study is the focus on malignant prostate lesions which can deliver bias. Benign lesions were excluded beforehand, since this study was not aimed to describe any possible overlap in semi-quantitative parameters between benign and malignant lesions. To our view further studies, preferably prospective with a large patient cohort, should be performed to evaluate possible differences in semi-quantitative parameters between benign and malignant prostate lesions.

A second limitation is the usage of two different scanner systems without separately evaluation since this study was not aimed at PET scanner evaluation.

## Conclusion

Two-time point [^68^Ga]Ga-PSMA-11 PET/CT demonstrated a detection rate of 94%. In primary tumor SUVmax values were increasing over the acquisition time. Higher PSA levels and higher Gleason grade group were associated with higher SUVmax levels in the primary tumor. Early imaging provides additional information in a small sub-group with declining semi-quantitative parameters in the late phase.

All data generated or analyzed are included in this published article and its supplementary information files. Further data on this study is available from the corresponding author on reasonable request.

## Abbreviations

PET/CT: Positron emission tomography/computed tomography
[^68^Ga]Ga-PSMA-11: Gallium-68 marked prostate specific membrane antigen
PCa: Prostate cancer
SUVmax: Standard uptake value maximum
SUVmean: Standard uptake value mean
TTV: Total tumor volume with PSMA uptake
TLU: Total lesion PSMA uptake
MBq: Mega-Becquerel
